# The Effectiveness of Ultrasound-Guided, Continuous, Bilateral Erector Spinae Plane Block in Perioperative Pain Management of Patients Undergoing Colorectal Surgery: A Randomized, Controlled, Double Blind, Prospective Trial

**DOI:** 10.3390/jcm12237465

**Published:** 2023-12-01

**Authors:** Freideriki Sifaki, Theodosia Vogiatzaki, Ioannis Mantzoros, Eleni Koraki, Panagiotis Christidis, Manousos-Georgios Pramateftakis, Vaia Tsapara, Stella Bagntasarian, Orestis Ioannidis, Pelagia-Paraskevi Chloropoulou

**Affiliations:** 1 Department of Anesthesiology, “Papageorgiou” General Hospital of Thessaloniki, 56429 Thessaloniki, Greece; fsifaki@med.duth.gr (F.S.); eleni.koraki@yahoo.gr (E.K.); 2 Department of Anesthesiology, Medical School, Democritus University of Thrace, General Hospital of Alexandroupolis, 68100 Alexandroupoli, Greece; tvogiatz@med.duth.gr (T.V.); peliachl@gmail.com (P.-P.C.); 3 4th Department of Surgery, Medical School, Aristotle University of Thessaloniki, “Georgios Papanikolaou” General Hospital of Thessaloniki, 57010 Thessaloniki, Greece; imanvol@gmail.com (I.M.); panagiotischristidis13@gmail.com (P.C.); mpramateftakis@hotmail.com (M.-G.P.); 4 Department of Anesthesiology, “Georgios Papanikolaou” General Hospital of Thessaloniki, 57010 Thessaloniki, Greece; vagia.tsap95@gmail.com (V.T.); green.volvox@gmail.com (S.B.)

**Keywords:** abdominal surgery, colorectal surgery, erector spinae plane block, regional anesthesia, perioperative pain management, postoperative analgesia, multimodal analgesia

## Abstract

Open and laparoscopic colorectal surgeries, while essential in the management of various colorectal pathologies, are associated with significant postoperative pain. Effective perioperative pain management strategies remain an anesthesiologic challenge. The erector spinae plane block (ESPB), a novel peripheral nerve block, has gained attention for its potential in providing analgesia for a wide variety of surgeries. This study aimed to evaluate the effectiveness of continuous, bilateral ultrasound-guided ESPB in perioperative pain management of patients undergoing colectomy. This prospective, randomized, controlled, double-blind trial included 40 adult patients scheduled for elective open or laparoscopic colectomy. Patients undergoing open colectomy as well as patients undergoing laparoscopic colectomy were randomly allocated into two groups: the ESPB group (*n* = 20) and the control group (*n* = 20). All patients received preoperatively ultrasound-guided, bilateral ESPB with placement of catheters for continuous infusion. Patients in the ESPB group received 0.375% ropivacaine, while patients in the control group received sham blocks. All patients received standardized general anesthesia and multimodal postoperative analgesia. Pain scores, perioperative opioid consumption, and perioperative outcomes were assessed. Patients in the ESPB group required significantly less intraoperative (*p* < 0.001 for open colectomies, *p* = 0.002 for laparoscopic colectomies) and postoperative opioids (*p* < 0.001 for open colectomies, *p* = 0.002 for laparoscopic colectomies) and had higher quality of recovery scores on the third postoperative day (*p* = 0.002 for open and laparoscopic colectomies). Patients in the ESPB group did not exhibit lower postoperative pain scores compared to those in the control group (*p* > 0.05 at various time points), while patients in both groups reported comparable satisfaction scores with their perioperative pain management (*p* = 0.061 for open colectomies, and *p* = 0.078 in laparoscopic colectomies). No complications were reported. ESPB is a novel and effective strategy in reducing perioperative opioid consumption in patients undergoing colectomy. This technique, as part of a multimodal analgesic plan and enhanced recovery after surgery protocols, can be proven valuable in improving the comfort and satisfaction of patients undergoing colorectal surgery.

## 1. Introduction

Colorectal surgery remains the therapeutic cornerstone for the management of a wide variety of gastrointestinal disorders encompassing malignancies and inflammatory conditions. Over the years, surgical interventions in the colorectal domain have advanced, including laparoscopic methods, and they are offering the promise of improved patient outcomes and enhanced quality of recovery [1]. However, effective goal-directed pain management in the perioperative period continues to pose a challenge for both patients and clinicians.

Pain during and following colorectal surgery often necessitates the use of opioids for numerous days, which, while effective, are associated with a spectrum of undesirable side effects, including respiratory depression and respiratory complications, sedation, nausea, and delayed postoperative mobilization of the patients and of their gastrointestinal tract [2]. Moreover, modern anesthesia practices tend to limit the opioids administered to patients due to potential opioid-related complications, such as tolerance and dependency, and due to the “opioid crisis” observed in many countries globally [3]. Thus, the quest for novel and more refined analgesic techniques has led to the implementation of regional anesthesia practices aimed at mitigating patients’ postoperative pain and reducing the systemic administration of opioids.

Erector spinae plane block (ESPB) is a novel trunk block, initially described by Forero et al. in 2016 for the relief of chronic neuropathic pain [4]. Since then, it has gained prominence as a regional anesthesia technique with the potential to revolutionize postoperative pain management, and it has been effectively administered not only for the management of perioperative pain for a wide variety of surgeries [5] but also for the management of acute post-traumatic pain [6] and chronic pain [7]. While the ESPB has exhibited efficacy in various surgical and nonsurgical procedures, its application and efficacy in colorectal surgery remains an area of growing interest.

This prospective, randomized, controlled, double-blind trial is designed to assess the effectiveness of ultrasound-guided, continuous, bilateral ESPB in perioperative pain management of patients undergoing colorectal surgery. Our study aims to assess the impact of ESPB on perioperative opioid consumption, postoperative pain scores, various postoperative aspects of patients’ recovery and ESPB-associated complications. Furthermore, this study aims to explore patient-reported outcomes, including quality of recovery and overall satisfaction, in order to comprehensively evaluate the utility of this regional anesthetic technique in enhancing the recovery experience of patients undergoing open or laparoscopic colectomy.

## 2. Materials and Methods

### 2.1. Study Design

This prospective study, conducted at the “Georgios Papanikolaou” General Hospital in Thessaloniki, Greece, employed a randomized, controlled, double-blinded design. Ethical approval (No: 1147/7.10.2019, October 2019) was obtained from the Local Ethics Committee, and the study adhered to the principles of the Declaration of Helsinki. The blinding protocol extended to patients, surgeons, anesthesiologists, operating theater staff, and surgical ward nurses. Recruitment occurred between January 2020 and August 2023, with all participants providing written informed consent. This study has been registered on clinicaltrials.gov with the reference number NCT04879004.

### 2.2. Inclusion and Exclusion Criteria

The research involved a cohort of 40 participants, encompassing both males and females, with ages ranging from 35 to 85 years. These individuals were categorized under the American Society of Anesthesiologists (ASA) physical status classes I, II, and III. The study focused on patients who underwent elective open or laparoscopic colectomy, performed by an experienced team of general surgeons. Exclusions from the study criteria comprised individuals who declined to provide consent, those classified as ASA > III, individuals transferred to the intensive care unit (ICU) postsurgery, and those with contraindications for regional anesthesia. Additionally, individuals with severe kidney or liver disease, along with those with known psychiatric disorders or a history of drug/alcohol abuse, were not included in this research.

### 2.3. Study Groups

The study included four groups:

(1) In Group CO (control group—open colectomy), ESPB was performed on the participants under ultrasound guidance, bilaterally, at T10 level with 40 mL of N/S 0.9% (20 mL on each side).
(2) In Group BO (block group—open colectomy), ESPB was performed on the participants under ultrasound guidance, bilaterally, at T10 level with 40 mL of ropivacaine 0.375% (20 mL on each side).
(3) In Group CL (control group—laparoscopic colectomy), ESPB was performed on the participants under ultrasound guidance, bilaterally, at T10 level with 40 mL of N/S 0.9% (20 mL on each side).
(4) In Group BL (block group—laparoscopic colectomy), ESPB was performed on the participants under ultrasound guidance, bilaterally, at T10 level with 40 mL of ropivacaine 0.375% (20 mL on each side).

The allocation and randomization of patients were carried out using the sealed envelope method and computer-generated random numbers.

### 2.4. Anesthesia

Before surgery, a uniform clinical examination of all participants was conducted by the same team of anesthesiologists, and standard preoperative laboratory tests were carried out.

**Figure 1:**
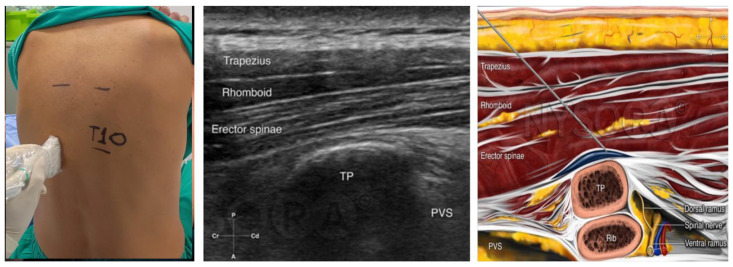
Erector spinae plane block. The transducer is placed in transverse position on the preferred spinous process, and 3 cm laterally, the transverse process of the vertebra that is selected is identified. The transducer is turned sagittally, and the landmarks (trapezius muscle, rhomboid muscle, erector spinae muscle, and transverse process) are identified. The needle is inserted in a cephalad-to-caudad orientation and an in–plane technique is performed. The needle is advanced until its tip reaches the fascia between the transverse process and the erector spinae muscle and the local anesthetic is administered [8]. The figure was reprinted with permission from source: NYSORA.COM.

Bilateral erector spinae plane block (ESPB) was administered to all participants while in the seated position, 30 min before the initiation of general anesthesia. This procedure was performed under ultrasound guidance, utilizing a linear transducer (7–13 MHz) and an 80 mm, 22-gauge, short bevel needle. Initially the transducer was placed transversely on the spinous process of T10, and 3 cm laterally, the transverse process of T10 was identified. Subsequently, the transducer was repositioned sagittally, and the correct landmarks were identified. The needle was then inserted in a cephalad-to-caudad orientation, employing an in-plane technique. The needle was advanced, and 20 mL of the prepared solution was administered in the fascia between the transverse process and the erector spinae muscle, at each side. After completion of the block, a catheter for the continuous infusion of local anesthetic was inserted on each side (see Figure 1).

Intraoperative monitoring of the patient included oximetry, invasive and noninvasive arterial blood pressure, electrocardiography, and BIS monitor. Furthermore, the intraoperative perception of patients’ pain was monitored via NOL monitor readings and the cardiac output/stroke volume variation of each patient was continuously recorded intraoperatively via Vigileo monitor readings. The initiation of general anesthesia involved administering fentanyl (1 mcg/kg), propofol (2 mg/kg), and rocuronium (0.6 mg/kg), following a 5 min preoxygenation period of the patient. Continuous maintenance of general anesthesia was achieved through titration of desflurane, guided by BIS readings with a target range of 40–60. Intraoperatively, titration of remifentanil was dictated by objective NOL readings along with patients’ vital signs. After tracheal intubation, the remifentanil infusion rate was set to 0.05 μg/kg/min. The rate was increased to 0.2 μg/kg/min one minute before skin incision. After skin incision, the infusion of remifentanil was reduced by 0.03 μg/kg/min when NOL readings were under 10 for more than one minute. The infusion of remifentanil was increased by 0.03 μg/kg/min when NOL readings were over 25 for more than one minute, followed by a bolus dose of remifentanil 30 μg. The NOL index was reassessed after 5 min when changing the infusion rate of remifentanil. All patients received intravenously MgSO_4_ 50 mg/kg after induction of general anesthesia. Additionally, 30 min prior to the conclusion of surgery, patients received paracetamol (1000 mg) and tramadol (100 mg). At the stage of placing the final sutures, a train-of-four (TOF) test was conducted to evaluate the extent of neuromuscular blockade, and sugammadex was administered as necessary for the reversal of neuromuscular blockade. Patients were discharged from postanesthesia care unit (PACU) when they achieved a score > 8 on the Aldrete’s recovery scale. Postoperatively, all patients received intravenously paracetamol 1000 mg every 6 h, and if their pain was assessed with a score > 4 on the numerical rating scale, they were offered tramadol 100 mg. If the reported pain was unbearable, the protocol of the study included the administration of intravenous morphine. Postoperatively and for the first 48 h, every 12 h 20 mL of ropivacaine 0.2% or N/S 0.9% (depending on the group to which the patient was allocated) was administered through the continuous infusion catheters at the site of the ESPB performance. After 48 h, the catheters were removed.

The study involved open and laparoscopic colectomies conducted by a consistent and skilled surgical team. The administration of erector spinae plane block (ESPB) to each patient was performed by the same proficient anesthesiologist.

### 2.5. Outcomes and Statistical Analysis

The main objective of this study was to determine variations in the overall postoperative tramadol consumption among the groups. Secondary outcomes included discrepancies in total intraoperative remifentanil usage, duration of stay in the postanesthesia care unit (PACU), NRS pain scores at different intervals following surgery completion, occurrences of postoperative nausea and vomiting (PONV), time until the initial patient mobilization and gastrointestinal tract activity, timing of the first intake of oral fluids and nutrition, along with hospitalization days. This study also explored the differences in patient-reported outcomes between the groups, including quality of recovery at the third postoperative day and overall satisfaction of the patients regarding their postoperative pain management.

The sample size for the open colorectal surgeries was based on the first 24 h tramadol requirement of patients undergoing open colorectal cancer surgery. To calculate the sample size, a clinically significant reduction in 24 h tramadol consumption was considered a 50% reduction. Initial pilot studies of 5 patients revealed that the mean first 24 h tramadol consumption was 340.0 ± 89.4 mg for the control group. According to the power analysis with independent samples *t*-test, using an electronically available one (https://clincalc.com/stats/samplesize.aspx, accessed on 1 March 2020), the minimum number of patients to be included in the study according to a confidence interval of 95% (1 − α), a test power of 80% (1 − β), a sampling ratio of 1, and a one-way hypothesis was determined to be 4 patients in each group. Taking into account the possibility of data loss or patient dropout, 10 patients were included in each group of patients undergoing open colectomy.

The sample size for the laparoscopic colorectal surgeries was based on the first 24 h tramadol requirement of patients undergoing laparoscopic colorectal cancer surgery. To calculate the sample size, a clinically significant reduction in 24 h tramadol consumption was considered a 50% reduction. Initial pilot studies of 5 patients revealed that the mean first 24 h tramadol consumption was 240.0 ± 89.4 mg for the control group. According to the power analysis with independent samples *t*-test, using an electronically available one (https://clincalc.com/stats/samplesize.aspx, accessed on 1 March 2020), the minimum number of patients to be included in the study according to a confidence interval of 95% (1 − α), a test power of 80% (1 − β), a sampling ratio of 1, and a one-way hypothesis was determined to be 9 patients in each group. Taking into account the possibility of data loss or patient dropout, 10 patients were included in each group of patients undergoing laparoscopic colectomy.

The measured variables were checked for the normality of their distribution by the Shapiro–Wilk test. Normally distributed, continuous variables were expressed by the arithmetic mean ± standard deviation (mean ± SD), while continuous variables with nonparametric distribution were expressed by median and intraquadratic range (median, IQR). Qualitative variables, categorical or ordinal, are presented as numbers and percentages per 100. The confidence interval was set at 95% which means that the differences between the groups were considered statistically significant when *p* < 0.05. To compare the variables in two independent study groups, a *t*-test was used for parametric distributable data, whereas Mann–Whitney U test was used for nonparametric distributable data. The comparisons of the independent nominal variables were performed with a chi-square test, as the expected counts were more than 5. The statistical analysis of the results was performed using the statistical program Jamovi Version 1. 6. 18.0.

Comparisons were performed between Group CO and Group BO and between Group CL and Group BL.

## 3. Results

**Figure 2:**
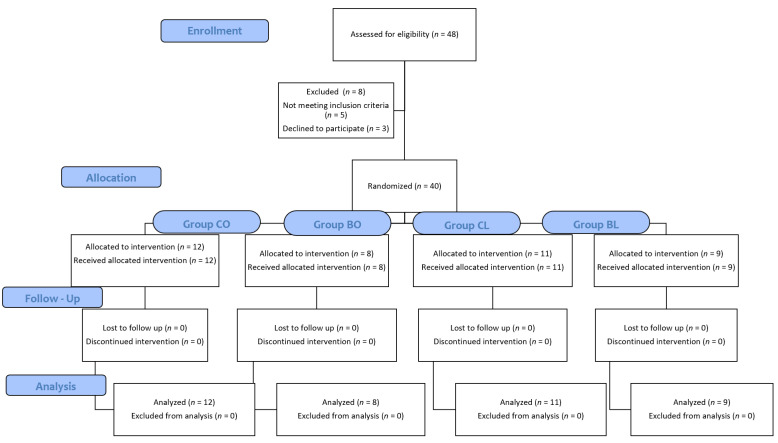
CONSORT flow diagram. Group CO (control group–open colectomy); Group BO (block group–open colectomy); Group CL (control group–laparoscopic colectomy); Group BL (block group–laparoscopic colectomy).

A total of 48 patients underwent evaluation for eligibility, with 40 meeting the criteria and participating in the study. Three patients declined participation, and five individuals did not meet the inclusion criteria. The inclusion and exclusion of patients are illustrated in the CONSORT flow diagram (Figure 2).

Patient demographic characteristics were similar among the groups (Table 1).

Throughout the surgical procedure and the hospital stay, all patients maintained hemodynamically stable, and there were no recorded complications related to the administration of erector spinae plane block (ESPB) or the surgical process.

The surgical time and the anesthesia time of the open and the laparoscopic colectomies were similar for the patients that were allocated to either the block or the control groups. On the contrary, the extubation time (time from the placement of the last surgical drapes until extubation) was statistically significantly shorter in patients that were allocated to the block groups when they were compared to the patients that were allocated to the control groups in both open (*p* = 0.004) and laparoscopic colectomies (*p* = 0.038) (Table 2).

Concerning the primary objective of this study, which is the postoperative tramadol consumption (mg), we observed a statistically significant difference in the total postoperative tramadol consumption between Groups CO and BO (*p* < 0.001) as well as between Groups CL and BL (*p* = 0.002). When administering morphine postoperatively and needing to determine its tramadol equivalent, we employed the following conversion factor: 1 mg of morphine equals 10 mg of tramadol [9]. Intraoperative remifentanil consumption was significantly lower in the BO and BL groups when compared to the CO and CL groups (*p* < 0.001 for open colectomies, *p* = 0.002 for laparoscopic colectomies) (Table 3).

NRS pain scores upon discharge from the PACU were found to be statistically significantly lower in the patients that were allocated to the BO and BL groups compared to the patients that were allocated to the CO and CL groups (*p* < 0.001 for open colectomies, *p* = 0.013 for laparoscopic colectomies). NRS pain scores at other various time points (12, 24, 36, 48, 60, 72, 84, and 96 h after completion of surgery) were not found to be statistically different between block groups and control groups, not only for the patients who underwent open colorectal surgery but also for the patients who underwent laparoscopic colorectal surgery (Table 4).

The median time spent in the postanesthesia care unit was notably lower in Group BO than in Group CO (*p* < 0.001). Similarly, Group BL exhibited a significantly reduced median PACU duration compared to Group CL (*p* = 0.011). Patients in Group BO demonstrated a significantly shorter time to mobilization compared to those in Group CO (*p* = 0.022). However, no such difference was observed when comparing patients in Group BL with those in Group CL (*p* = 0.492). Regarding the mobilization of the gastrointestinal tract, no statistically significant differences were found for open colectomies (*p* = 0.068) or laparoscopic colectomies (*p* = 0.09). Notably, the initiation of oral fluids and nutrition was significantly quicker in patients in Group BO compared to those in Group CO (pFluids = 0.01 and pNutrition = 0.014). This difference was statistically significant only for the start of oral fluids for the patients allocated to group BL when compared to the patients of group CL (pFluids = 0.002 and pNutrition = 0.068). The hospitalization days did not present a statistically important difference between the groups (*p* = 0.761 for open colectomies, *p* = 0.452 for laparoscopic colectomies) (Table 5).

No statistically significant difference was exhibited regarding PONV of patients 12, 24, 48, 60, 72, 84, and 96 h after completion of surgery among the groups studied (Table 6).

Patients’ quality of recovery was recorded at the third postoperative day, using a QoR-15 questionnaire and was found to be statistically significantly higher in patients who had received an ESPB with local anesthetic as compared to patients who received sham blocks (*p* = 0.002 for open colectomies and *p* = 0.019 for laparoscopic colectomies). The satisfaction score regarding patients’ postoperative analgesia management was comparable between groups (*p* = 0.061 for open colectomies, and *p* = 0.078 for laparoscopic colectomies) (Table 7).

## 4. Discussion

According to the results of this randomized, controlled, double-blinded, prospective trial, ultrasound-guided bilateral ESPB significantly mitigated the perioperative use of opioids in patients undergoing elective open or laparoscopic colectomy. More specifically, in groups BO and BL, total intraoperative remifentanil and postoperative tramadol consumption were found to be significantly lower when compared to groups CO and CL. ESPB proved to be effective not only in limiting the perioperative opioids administered to patients undergoing open and laparoscopic colectomy but also in shortening the time spent in the postanesthesia care unit for patients undergoing open and laparoscopic colectomy and the time until first ambulation after open colectomy. Interestingly, ESPB was found to be a key factor in ameliorating the quality of recovery of patients undergoing elective open or laparoscopic colorectal surgery.

Postoperative pain represents a pivotal determinant in the overall quality of patients’ postoperative recovery. This multifaceted factor of surgical recovery can potentially retard the patients’ ambulatory progress, augment their hospital stay, and increase the financial outlay incurred during their hospitalization. Moreover, it can significantly impede the patients’ overall contentment with the surgical experience. Thus, the cornerstone of modern anesthesia practices should be the provision of effective perioperative analgesia. Achieving this goal necessitates organizing a multimodal analgesic plan which includes different categories of analgesic agents and adjuvants, complemented by the implementation of regional anesthetic techniques.

Pain following open or laparoscopic colectomy surgery is a complex phenomenon, stemming from a variety of contributing factors, including visceral, somatic, and referred pain components. Notably, even in the context of laparoscopic colectomies, which are minimally invasive procedures, patients often report elevated numeric rating scale (NRS) scores indicative of significant pain experiences [10].

Over the previous years, a wide variety of different regional analgesic techniques have been tried to effectively address perioperative pain stemming from colorectal surgery. While the ERAS Society advises the utilization of epidural techniques as an analgesic strategy for open colorectal surgeries [1], the ideal method for managing pain during and after laparoscopic colorectal surgery continues to remain an unanswered question. A meta-analysis from Peltrini et al. suggests that transversus abdominis plane (TAP) block performance provides effective analgesia and a significant reduction in opioid administration on the first postoperative day after laparoscopic colorectal surgery [11]. Similarly, the performance of lateral quadratus lumborum block has proven to be effective in enhancing patients’ recovery after laparoscopic colorectal surgery and in the alleviation of their postoperative pain [12]. There have been trials exploring the efficacy of rectus sheath block in laparoscopic abdominal surgeries, however its analgesic efficacy did not surpass thoracic epidural analgesia [13].

Erector spinae plane block is an innovative analgesic approach, while its mechanism of action is still being investigated. As per the existing literature, during ESPB administration, local anesthetic diffuses through various fascial planes, exerting its anesthetic action on both the ventral and dorsal rami of spinal nerves. Some studies presume that local anesthetic disperses into various spaces, including paravertebral and epidural spaces. Thus, ESPB offers not only somatic but also visceral analgesia, and it can be implemented in multimodal analgesia protocols for a wide variety of surgeries [14].

In terms of potential complications associated with this block, studies have reported the occurrence of local anesthetic systemic toxicity (LAST) as well as weakness of the erector spinae muscle and pneumothorax occurrence [15]. In this study, the block was administered by the same proficient anesthesiologist to all patients, and no complications regarding the ESPB administration were reported.

In our study, we performed ESPB bilaterally under ultrasound guidance at the T10 level preoperatively in all patients, using 40 mL of N/S 0.9% (control groups) or ropivacaine 0.375% (block groups), and after the completion of the block, we inserted one catheter on each side, designed specifically for the administration of local anesthetic at the site of ESPB performance. Administering the ESPB preoperatively is crucial, as it offers the opportunity to the anesthesiologist to assess the efficacy of the block, to monitor any complications that may arise from the administration of ESPB, as well as to reduce the administration of opioids not only postoperatively but also intraoperatively. Across all existing studies in the current literature that investigate the effectiveness of bilateral erector spinae plane block (ESPB) in open and laparoscopic colectomies, the block was administered either before or after the induction of general anesthesia [16,17,18,19,20,21].

In the existing literature, ESPB is performed with the administration of bupivacaine, levobupivacaine, or ropivacaine in different concentrations (0.25% to 0.5%). In all studies of the literature that exists up until now, 20 mL of local anesthetic mixture is administered to the site of ESPB performance, at the T8, T9, or T10 level. In our study, 20 mL of ropivacaine 0.375% was administered at the T10 level. Ropivacaine was selected over bupivacaine as it has less cardiotoxic effects [22]. Moreover, in our study, catheters for the continuous infusion of local anesthetic were placed at the site of ESPB performance, and to the authors’ knowledge, this study represents the first investigation of the efficacy of continuous ESPB when performing this regional anesthesia technique on patients undergoing elective open or laparoscopic colectomy. These catheters provide an opportunity for the anesthesiologist to deliver local anesthetic to the ESPB site, even once the initial bolus dose of ropivacaine has worn off. This capability aids in postoperative pain management, extending its benefits beyond the first day after surgery.

Total median intraoperative remifentanil consumption was significantly lower in the BO and BL groups when compared with the CO and CL groups, respectively. The findings of this study align with the trials already documented in the literature [18,20]. Similarly, the total median postoperative tramadol consumption was significantly lower in both block groups when compared to the control groups, a result which aligns with the results of the studies that already exist in the literature and prove that ESPB administration can lower the postoperative administration of opioids in patients undergoing open or laparoscopic colectomy [16,17,18,19,20]. The mitigation of perioperative opioids is a fundamental finding, as opioids can have detrimental side effects related to patients’ recovery process, including postoperative nausea and vomiting, respiratory depression and respiratory complications, delay of patients’ mobilization, postoperative ileus, tolerance, and addiction [2].

The association between PONV and perioperative opioid use is well established. Nevertheless, our study identified no statistically significant variance in this parameter among the investigated groups. This finding may be attributed to the systematic administration of antiemetics to patients during the initial days following surgery.

The NRS scores of the patients were found to be statistically lower between the BO and BL groups versus the CO and CL groups only at discharge from the PACU, but not 12, 24, 36, 48, 60, 72, 84, and 96 h after surgery. This result may be explained by the methodology and the study design, which included the administration of intravenous tramadol 100 mg or morphine if the patient complained of postoperative pain with a score > 4 on the NRS.

Time spent in the postanesthesia care unit was less in the patients of the block groups when compared to control groups, a variable that has not been studied in the trials which exist in the current literature. This result implies that the administration of ESPB in patients undergoing elective open or laparoscopic colectomies can contribute to faster recovery after anesthesia and surgery.

Mobilization time of the patient was found to be significantly shorter in patients allocated to the BO group when they were compared to patients of the CO group; however, this result was not recorded among patients undergoing laparoscopic colectomy. This finding supports the idea that laparoscopic procedures, which are minimally invasive, present additional advantages, including early mobilization of patients after surgery, whereas patients who undergo open procedures may benefit from the application of a regional anesthetic technique.

There was no statistically significant difference reported regarding the satisfaction score of the patients about their postoperative analgesia between groups. Only one study in the existing literature explores and highlights ESPB’s dominance in augmenting patients’ satisfaction score regarding their postoperative analgesia [18]. This outcome can be rationalized by the research design, which ensured efficient postoperative pain management for all participants, regardless of their assignment to either the block or control groups. It included the administration of intravenous tramadol 100 mg or morphine if a patient reported postoperative pain with a NRS score > 4.

Regarding the duration of hospitalization of patients, we observed no noteworthy distinction between the study groups. This outcome can be attributed to the surgeons’ standardized practice of discharging patients on the fifth or sixth day following surgery provided there are no reported complications.

Regarding the patients’ perception of recovery, the QoR-15 questionnaire was used by all participants on the third postoperative day. Patients who were allocated to the block groups reported higher QoR scores when compared to patients in the control groups. In the current literature, only one clinical trial examines and validates that the quality of recovery for patients undergoing laparoscopic colorectal surgery is enhanced when erector spinae plane block (ESPB) is administered [19]. This finding is crucial, as it demonstrates the importance of ESPB application in order to achieve enhanced recovery after surgery in the context of open and laparoscopic colectomies.

One limitation of our study is the relatively modest size of the patient cohort included. This factor may have contributed to our inability to identify statistically significant variances concerning certain study variables. Another limitation of our study is that we assessed the static but not the dynamic pain scores of participants’ postoperative pain. When assessing dynamic pain scores, continuous assessments are needed, which was very challenging and was not feasible in the busy clinical environment of our hospital, which has limited resources and personnel. As such, we prioritized the collection of other outcome measures (pain scores at rest, total opioid requirements, time to first ambulation etc.).

In terms of the strengths of our study, every open and laparoscopic colectomy was performed by a skilled surgical team, and erector spinae plane block (ESPB) was administered to each patient by the same proficient anesthesiologist. Furthermore, the blinding protocol encompassed not only the patients but also the surgeons, anesthesiologists, operating theater personnel, and surgical ward nurses. ESPB was administered preoperatively, with the goal of evaluating its efficacy in perioperative pain management for individuals undergoing elective colorectal procedures. Furthermore, our study is the first, to our knowledge, to explore the use of continuous infusion catheters at the ESP administration site, enabling the delivery of local anesthetic boluses even after the completion of surgery. This unique approach motivated our current research endeavor.

## 5. Conclusions

Based on our study’s findings, it is evident that the incorporation of ESPB into multimodal analgesia protocols offers a distinct advantage in terms of perioperative opioid requirements. Additionally, it appears to reduce the time spent in the PACU and to enhance postoperative quality of recovery scores when compared to a placebo. ESPB represents a secure, innovative, and efficient methodology that can enhance the postoperative recovery of patients, following elective colorectal surgery.

The outcomes of this study, as well as the existing body of literature on ESPB in patients undergoing elective colorectal surgery, offer promising prospects. Undoubtedly, these findings should be further validated through additional randomized controlled trials. 

## Figures and Tables

**Table 1:** Patients’ demographic characteristics.

Variables		Group BO (*n* = 8)	Group CO (*n* = 12)	*p*-Value
Age, m ± sd (years)		64.6 ± 13.3	64.6 ± 11.2	0.994
Gender, *n*%	Female	3/8 (37.5%)	7/12 (58.3%)	0.361
	Male	5/8 (62.5%)	5/12 (41.7%)	
BMI, m ± sd (kg/m^2^)		30.3 ± 7.14	25.9 ± 3.6	0.085
ASA, *n*%	ASA 2	3/8 (37.5%)	7/12 (58.3%)	0.361
	ASA 3	5/8 (62.5%)	5/12 (41.7%)	
NYHA, *n*%	NYHA 1	3/8 (37.5%)	4/12 (33.3%)	0.848
	NYHA 2	5/8 (62.5%)	8/12 (66.7%)	
**Variables**		**Group BL** **(*n* = 9)**	**Group CL** **(*n* = 11)**	***p*-Value**
Age, m ± sd (years)		66.7 ± 9.11	71.4 ± 10.4	0.321
Gender, *n*%	Female	3/9 (33.3%)	4/11 (36.4%)	0.888
	Male	6/9 (66.7%)	7/11 (63.6%)	-
BMI, m ± sd (kg/m^2^)		26.7 ± 2.84	28.7 ± 3.93	0.230
ASA, *n*%	ASA 1	3/9 (33.3%)	5/11 (45.5%)	0.582
	ASA 2	6/9 (66.7%)	6/11 (54.5%)	
NYHA, *n*%	NYHA 1	3/9 (33.3%)	3/11 (27.3%)	0.769
	NYHA 2	6/9 (66.7%)	8/11 (72.7%)	

m: mean; sd: standard deviation; BMI: body mass index; kg: kilogram; m^2^: meters^2^; ASA: American Society of Anesthesiologists; NYHA: New York Heart Association.

**Table 2:** Surgical time and extubation time.

Variables	Group BO (*n* = 8)	Group CO (*n* = 12)	*p*-Value
Surgical time, m ± sd (min)	175.3 ± 55.7	136.7.0 ± 40.6	0.089
Anesthesia time, m ± sd (min)	182.3 ± 30.3	151.8 ± 33.6	0.054
Extubation time, median (IQR) (min)	2.5 (3.0)	6.5 (6.25)	0.004
**Variables**	**Group BL** (***n* = 9)**	**Group CL** **(*n* = 11)**	***p*-Value**
Surgical time, m ± sd (min)	168.0 ± 62.8	148.0 ± 50.1	0.594
Anesthesia time, m ± sd (min)	206.0 ± 68.6	192.0 ± 53.0	0.849
Extubation time, median (IQR) (min)	2.0 (4.0)	10.0 (10.5)	0.038

m: mean; sd: standard deviation; IQR: interquartile; min: minutes.

**Table 3:** Perioperative opioid consumption.

Variables	Group BO (*n* = 8)	Group CO (*n* = 12)	*p*-Value
Remifentanil, m ± sd (mcg)	440.0 ± 468.0	1318.0 ± 362.0	<0.001
Tramadol (mg), m ± sd	158.0 ± 73.3	686.0 ± 422.0	<0.001
**Variables**	**Group BL** **(*n* = 9)**	**Group CL** **(*n* = 11)**	***p*-Value**
Remifentanil, m ± sd (mcg)	394.0 ± 342	1177.0 ± 476	0.002
Tramadol (mg), m ± sd	139.0 ± 41.7	508.0 ± 338.0	0.002

m: mean; sd: standard deviation; mcg: microgram; mg: milligram.

**Table 4:** NRS pain scores.

Variables	Group BO (*n* = 8)	Group CO (*n* = 12)	*p*-Value
NRS on discharge from PACU, median (IQR)	0.0 (0.25)	2.0 (0.5)	<0.001
NRS on rest 12 h, median (IQR)	0.0 (3.0)	2.5 (3.25)	0.273
NRS on rest 24 h, median (IQR)	0.0 (2.0)	1.5 (3.0)	0.186
NRS on rest 36 h, median (IQR)	0.0 (1.25)	0.5 (3.0)	0.458
NRS on rest 48 h, median (IQR)	0.0 (0.25)	0.0 (1.0)	0.738
NRS on rest 60 h, median (IQR)	0.0 (0.0)	0.0 (0.0)	0.852
NRS on rest 72 h, median (IQR)	0.0 (0.0)	0.0 (0.0)	0.882
NRS on rest 84 h, median (IQR)	0.0 (0.0)	0.0 (0.0)	0.938
NRS on rest 96 h, median (IQR)	0.0 (0.0)	0.0 (0.0)	0.456
**Variables**	**Group BL** **(*n* = 9)**	**Group CL** **(*n* = 11)**	***p*** **-Value**
NRS on discharge from PACU, median (IQR)	0.0 (0.0)	3.0 (3.5)	0.013
NRS on rest 12 h, median (IQR)	1.0 (3.0)	2.0 (1.0)	0.394
NRS on rest 24 h, median (IQR)	1.0 (3.0)	0.0 (2.0)	0.379
NRS on rest 36 h, median (IQR)	1.0 (2.0)	2.0 (1.5)	0.377
NRS on rest 48 h, median (IQR)	0.0 (0.0)	0.0 (3.0)	0.066
NRS on rest 60 h, median (IQR)	0.0 (0.0)	1.0 (1.5)	0.013
NRS on rest 72 h, median (IQR)	0.0 (0.0)	0.0 (1.0)	0.112
NRS on rest 84 h, median (IQR)	0.0 (0.0)	0.0 (0.0)	0.374
NRS on rest 96 h, median (IQR)	0.0 (0.0)	0.0 (0.0)	0.374

PACU: postanesthesia care unit; IQR: interquartile; NRS: numerical rating scale.

**Table 5:** Times.

Variables	Group BO (*n* = 8)	Group CO (*n* = 12)	*p*-Value
PACU duration, m ± sd (min)	14.2 ± 2.89	40.0 ± 20.2	<0.001
Mobilization time, median (IQR) (h)	24.0 (22.5)	42.0 (12.0)	0.022
Mobilization of GIT time, m ± sd (h)	16.9 ± 8.02	24.4 ± 8.55	0.068
Start fluids, m ± sd (h)	19.0 ± 10.5	34.5 ± 13.5	0.010
Start enteral nutrition, m ± sd (h)	36.2 ± 12.7	51.0 ± 10.6	0.014
Discharge time, m ± sd (d)	6.25 ± 3.14	5.88 ± 1.64	0.761
**Variables**	**Group BL** **(*n* = 9)**	**Group CL** **(*n* = 11)**	***p*** **-Value**
PACU duration, m ± sd (min)	15.6 ± 1.67	22.2 ± 6.81	0.011
Mobilization time, median (IQR) (h)	24.0 (0.0)	24.0 (13.0)	0.492
Mobilization of GIT time, m ± sd (h)	13.1 ± 4.14	19.5 ± 9.88	0.090
Start fluids, m ± sd (h)	13.3 ± 4.0	32.0 ± 15.3	0.002
Start enteral nutrition, m ± sd (h)	32.0 ± 12.4	44.7 ± 16.2	0.068
Discharge time, m ± sd (d)	4.56 ± 1.24	5.09 ± 1.76	0.452

PACU: postanesthesia care unit; m: mean; sd: standard deviation; IQR: interquartile; min: minutes; GIT: gastrointestinal tract; h: hours; d: days.

**Table 6:** Postoperative nausea and vomiting.

Hours	Variable		Group BO (*n* = 8)	Group CO (*n* = 12)	*p*-Value
Discharge from PACU	POVN, *n*%	Yes	0/8 (0.0%)	0/12 (0.0%)	NA
12	POVN, *n*%	Yes	0/8 (0.0%)	1/12 (8.3%)	0.402
24	POVN, *n*%	Yes	0/8 (0.0%)	0/12 (0.0%)	NA
36	POVN, *n*%	Yes	1/8 (12.5%)	1/12 (8.3%)	0.761
48	POVN, *n*%	Yes	0/8 (0.0%)	0/12 (0.0%)	NA
60	POVN, *n*%	Yes	1/8 (12.5%)	0/12 (0.0%)	0.209
72	POVN, *n*%	Yes	0/8 (0.0%)	0/12 (0.0%)	NA
84	POVN, *n*%	Yes	0/8 (0.0%)	0/12 (0.0%)	NA
96	POVN, *n*%	Yes	0/8 (0.0%)	0/12 (0.0%)	NA
**Hours**	**Variable**		**Group BL** **(*n* = 9)**	**Group CL** **(*n* = 11)**	***p*-Value**
Discharge from PACU	POVN, *n*%	Yes	0/9 (0.0%)	1/11 (9.1%)	0.353
12	POVN, *n*%	Yes	0/9 (0.0%)	2/11 (18.2%)	0.178
24	POVN, *n*%	Yes	0/9 (0.0%)	0/11 (0.0%)	NA
36	POVN, *n*%	Yes	0/9 (0.0%)	4/11 (36.5%)	0.129
48	POVN, *n*%	Yes	0/9 (0.0%)	2/11 (18.2%)	0.178
60	POVN, *n*%	Yes	0/9 (0.0%)	2/11 (18.2%)	0.178
72	POVN, *n*%	Yes	0/9 (0.0%)	2/11 (18.2%)	0.178
84	POVN, *n*%	Yes	0/9 (0.0%)	1/11 (9.1%)	0.303
96	POVN, *n*%	Yes	1/9 (11.1%)	0/11 (0.0%)	0.303

PACU: postanesthesia care unit; PONV: postoperative nausea and vomiting; NA: not applicable.

**Table 7:** Quality of recovery and satisfaction score.

Variables	Group BO (*n* = 8)	Group CO (*n* = 12)	*p*-Value
Satisfaction score, median (IQR)	6.0 (1.0)	5.0 (0.25)	0.061
QoR 6, median (IQR)	145.0 (3.5)	135.0 (6.0)	0.002
**Variables**	**Group BL** **(*n* = 9)**	**Group CL** **(*n* = 11)**	***p*** **-Value**
Satisfaction score, median (IQR)	6.0 (0.0)	5.0 (1.0)	0.078
QoR 6, median (IQR)	143.0 (3.0)	139.0 (2.0)	0.019

QoR: quality of recovery; IQR: interquartile.

## Data Availability

Data are unavailable due to privacy restrictions.
